# Comparing simulated aerial and chemigation insecticide applications to manage western bean cutworm (Lepidoptera: Noctuidae) in corn

**DOI:** 10.1093/jee/toae306

**Published:** 2025-01-13

**Authors:** Andrea Rilaković, Alisson da Silva Santana, Miloš Zarić, Vamsi Manthena, Jeffrey A Golus, Greg R Kruger, Ana M Vélez, Julie A Peterson

**Affiliations:** University of Nebraska-Lincoln, West Central Research, Extension & Education Center, North Platte, NE, USA; University of Nebraska-Lincoln, West Central Research, Extension & Education Center, North Platte, NE, USA; University of Nebraska-Lincoln, West Central Research, Extension & Education Center, North Platte, NE, USA; Department of Statistics, University of Nebraska-Lincoln, Lincoln, NE, USA; University of Nebraska-Lincoln, West Central Research, Extension & Education Center, North Platte, NE, USA; University of Nebraska-Lincoln, West Central Research, Extension & Education Center, North Platte, NE, USA; Department of Entomology, University of Nebraska-Lincoln, Lincoln, NE, USA; University of Nebraska-Lincoln, West Central Research, Extension & Education Center, North Platte, NE, USA

**Keywords:** *Striacosta albicosta*, aerial application, chemigation, bifenthrin, chlorantraniliprole

## Abstract

The efficacy of aerial application and chemigation of insecticides is not well explored for western bean cutworm, *Striacosta albicosta* (Smith), management in corn. In the short term, inadequate application of insecticides can lead to control failures when insect pests are not effectively targeted. In the longer term, exposure to sublethal insecticide concentrations can contribute to the evolution of insecticide resistance. The goal of this study was to compare aerial application and chemigation under simulated conditions to determine the most effective insecticide application method for managing *S. albicosta*. Three larval stages were exposed to the highest and lowest label rates of commercial formulations of bifenthrin (36.8 and 112.1 g a.i. ha^−1^) and chlorantraniliprole (52.7 and 75.1 g a.i. ha^−1^). Experiments were conducted in spray chambers, utilizing a carrier volume of 18.7 L ha^−1^ for aerial application and 1.57 cm ha^−1^ for chemigation. Mortality was recorded at 16, 24, and 41 h after infestation. The simulated aerial application was more effective than simulated chemigation in controlling *S. albicosta*, resulting in 100% mortality 24 h after infestation. Within the chemigation applications, chlorantraniliprole treatments were effective at both rates for all instars, while the high rate of bifenthrin exhibited greater efficacy than the lower rate. In conclusion, it was evidenced that the same insecticide active ingredients do not yield equivalent efficacy when applied via aerial application compared to chemigation. The present study highlights the importance of selecting appropriate insecticide application methods in controlling *S. albicosta* larvae.

## Introduction

Insecticide applications targeting various pests in fully grown corn can be performed by aerial application and chemigation. Understanding their respective efficacies and limitations proves crucial for effective pest management, particularly for insect pests that rely upon insecticide applications as a part of an integrated pest management strategy. Aerial application ranks among the most common methods for insecticide application in corn fields during the tasseling stage due to its ability to efficiently cover vast areas. Around 127 million acres of cropland in the United States are annually sprayed via aerial application (Moore 2019). This method significantly surpasses the traveling velocity of alternative approaches such as ground application or chemigation. Its superior speed ensures timely intervention during critical growth stages of crops and maximizes productivity by minimizing potential yield losses due to pests. However, despite its efficiency, the spray coverage achieved through aerial application is not uniform across the entire corn canopy (Souza et al. 2019). For instance, Bynum et al. (1991) demonstrated that aerial spray deposition on flag, ear, and bottom leaves of the corn canopy were, on average 53%, 30%, and 17%, respectively. Chemigation is another important technique that involves the application of agrochemicals or pesticides through overhead irrigation systems such as central pivots or linear irrigation systems. One of the benefits of using this application method is that growers can use existing irrigation systems without additional costs to distribute insecticides in corn fields, thereby controlling insect pests (Chalfant and Young 1982). Other advantages of chemigation application over aerial application is the lower cost of the application, and the reduced exposure to humans (Chalfant and Young 1982, Dale Threadgill 1985). One significant difference between chemigation and any other application method is the volume of water that is used during application. Wei et al. (2017) reported that overhead irrigation pivot systems at the maximum speed would need a 300-fold higher water volume than the ground spray application per hectare. However, Geary et al. (2004) showed that using a lower amount of water for chemigation application might result in a more even distribution of applied pesticide through the canopy than other application techniques. In addition, applying a greater amount of water volume increases the risk of runoff onto the ground if it is not applied correctly (Cunha da and Nascimento 2013, EPA 1987).

Aerial and chemigation applications have been used to target the western bean cutworm, *Striacosta albicosta* (Smith) (Lepidoptera: Noctuidae), an economically important pest of corn (*Zea mays* L.) and dry beans (*Phaseolus vulgaris* L.) in the western Great Plains and Great Lakes regions of the United States and Canada (Archibald et al. 2018, Smith et al. 2018). This pest overwinters underground in a prepupal stage, preferably in sandy soils and completes 1 generation per year. In Nebraska, moths emerge in early- to mid-July and remain active until mid-August (Hagen 1962, Seymour et al. 2010). After mating, females lay eggs on the upper surfaces of corn leaves near the tassel (Smith et al. 2019). Upon hatching, the neonates migrate upward to feed on tassel tissue and pollen until reaching the third or fourth instar, when they then move down to feed on silk and developing kernels. Older larvae might enter the corn ear or move to neighboring corn plants within or across rows to feed (Paula-Moraes et al. 2012a, Pannuti et al. 2016). Given their unique feeding behavior, just one *S. albicosta* larva per plant in a corn field with a density of 74,100 plants per hectare could lead to an average yield loss of 945.52 kg per hectare (Paula-Moraes et al. 2013).

Integrated pest management tactics for *S. albicosta* in the western Great Plains mainly rely on use of transgenic *Bacillus thuringiensis* (Bt) corn and the application of insecticides after an economic threshold has been reached (Archibald et al. 2018). *S. albicosta* has developed resistance to the Cry1F protein (Smith et al. 2017, Coates et al. 2020), leaving Vip3A as the only remaining effective protein for management of this pest, although there are concerns about resistance evolution to the Vip3A protein (Farhan et al. 2018, 2019).

Consequently, reliance on transgenic corn for *S. albicosta* control is not always feasible primarily because corn hybrids expressing Vip3A have limited commercial availability in Nebraska’s market. When effective Bt proteins are absent, foliar insecticide applications are key for managing first, second, and third instar larvae. This approach takes advantage of *S. albicosta* larval dispersal behavior exposing younger instars to a higher insecticide dose prior to entering the corn ear (Seymour et al. 2010, Farhan et al. 2022). Current management recommendations advise applying insecticides when the economic threshold is reached: in Nebraska, this ranges from 5% to 8% of plants infested with egg masses or larvae of *S. albicosta* (Paula-Moraes et al. 2013). Application timing is recommended when 95% of the corn plants have tasseled. This approach aligns insecticide use with the pest’s life cycle and the corn’s growth stage, optimizing the timing for maximum effectiveness.

Pyrethroids (IRAC Class 3A) are sodium channel modulators and the most common class (81% of foliar insecticides) used to target *S. albicosta* in Nebraska (Archibald et al. 2018). They are favored due to their low cost and their ability to control other corn pests concurrently (Souza et al. 2019). Diamides (IRAC Class 28) (e.g., chlorantraniliprole) which are modulators of the ryanodine receptor, are a more recent chemistry than pyrethroids and offer another option for *S. albicosta* management. Chlorantraniliprole has the longest residual activity on corn plants for *S. albicosta* control compared to pyrethroids, spinosyn, and diacylhydrazine (Farhan et al. 2022). This residual activity might play a key role for the successful management of *S. albicosta,* especially if additional oviposition occurs after insecticide application. The efficacy of various foliar-applied insecticides have been examined (Montezano et al. 2017a, b, 2019a, Swoboda-Bhattarai et al. 2018, 2019a, b, Farhan et al. 2022, DeVries and Wright 2024). Still, the rotation of different modes of action for *S. albicosta* management and other corn pests has not been effectively practiced in recent years (Archibald et al. 2018).

Currently, the only available research on the efficacy of chemigation against *S. albicosta* dates back to 1984 (Pilcher and Peairs 1984), rendering it outdated due to the removal of tested products from the market and the obsolescence of the equipment. Grower and crop consultant reports have indicated dissatisfaction with the performance of pyrethroid insecticides against *S. albicosta* across various locations in Nebraska since 2014 (Archibald et al. 2018). While these field failures have raised concerns related to *S. albicosta* resistance, some farmers regularly employ chemigation for insecticide application despite limited research on its efficacy against *S. albicosta.* Studies in 2016 and 2017 reported low levels of bifenthrin resistance in Nebraska, suggesting that resistance might not be the only factor causing control failures (Montezano et al. 2019a). Instead, inadequate application methods could contribute to these failures and should be further investigated.

We conducted an experimental study within a controlled environment to assess the efficacy of insecticides applied via aerial application and chemigation for managing *S. albicosta*. Our hypotheses were that the application method, formulated product, and application rate would influence *S. albicosta* control. The study aimed to i) compare the performance of aerial application and chemigation methods under controlled conditions, and ii) investigate the efficacy of commercially formulated insecticides on the first 3 larval instars of *S. albicosta*.

## Materials and Methods

### *Striacosta albicosta* Collection and Rearing

Moths were collected from the West Central Research, Extension and Education Center (WCREEC) near North Platte, NE (41.085390, −100.773589) and the West Central Water Resources Field Laboratory near Brule, NE (41.158810, −102.026874) during 2021. Previous research indicated that there were no notable differences in *S. albicosta* susceptibility to Cry1F Bt toxin (Coates et al. 2020) or bifenthrin (Montezano et al. 2019a) between these 2 locations. Moths were collected using a trapping system previously described by Montezano et al. (2019b) and Coates et al. (2020). This system consisted of wooden field cages (dimensions of 117 × 120 × 220 cm) covered by metal screening with a black light trap on top of the cage. Pinto bean plants (*P. vulgaris*) were maintained inside each cage and used as a substrate for oviposition. During the moth flight, cages were checked every 2 days. The collected moths were placed in rearing cages (63.5 × 63.5 × 63.5 cm; Bug Dorm, MegaView Science Co., Ltd., Taichung, Taiwan) at the Agroecosystems Entomology Laboratory. Each rearing cage contained an adult diet consisting of a 5% sucrose and 0.2% ascorbic acid solution provided in a 150 mm diameter × 15 mm sponge inside a Petri dish (Montezano et al. 2019b). The rearing cages were monitored daily and maintained under natural lighting conditions with a room temperature of 26.6 ± 1 °C.

The eggs collected from field and rearing cages were placed on moistened filter paper inside plastic containers (Tupperware) and kept in Percival E-36HO growth chambers under controlled temperature (26.6 ± 1 °C), with relative humidity maintained between 70% and 80%, under a 16:8 (L:D) hour photoperiod (Montezano et al. 2019b). After hatching, larvae were fed with an artificial diet following Dyer et al. (2013) until they reached the desired instar. The developmental stage was tracked daily by observing shed head capsules (Montezano et al. 2019b), a common method used to monitor the development of larval instars of Lepidoptera species.

### Simulation of Aerial and Chemigation Application under Controlled Conditions

Simulation of aerial and chemigation applications was carried out at the Pesticide Application Technology Laboratory located at WCREEC during the summer of 2021. Ten treatments with 4 replications were tested. The pyrethroid insecticide bifenthrin (Brigade 2EC, FMC Corporation, Philadelphia, PA) was tested at the lowest 36.8 g a.i. ha^−1^ (2.1 fl oz acre^−1^), and the highest 112.1 g a.i. ha^−1^ (6.4 fl oz acre^−1^) label rates recommended for *S. albicosta* control in field corn applied to simulate both aerial and chemigation applications (Table 1). The diamide, chlorantraniliprole (Prevathon, FMC Corporation, Philadelphia, PA), was tested at the lowest 52.7 g a.i. ha^−1^ (14 fl oz acre^−1^) and the highest 75.1 g a.i. ha^−1^ (20 fl oz acre^−1^) label rates applied to simulate both aerial and chemigation application. Insecticide solutions were prepared in tap water at a rate of 18.7 L ha^−1^ (2 gallons acre^−1^) for aerial application and 1.57 cm-ha (0.25 ac-inch) for chemigation application. A control treatment for both application methods consisted of water only.

**Table 1.** Application types, products, active ingredients, and insecticide rates for aerial and chemigation applications for *Striacosta albicosta* control

Application type	Product	Active ingredients	Insecticide rate (g a.i. ha^−1^)
Aerial	Control	Water	–
Aerial	Prevathon	Chlorantraniliprole	52.7 (Low)
Aerial	Prevathon	Chlorantraniliprole	75.1 (High)
Aerial	Brigade	Bifenthrin	36.8 (Low)
Aerial	Brigade	Bifenthrin	112.1 (High)
Chemigation	Control	Water	–
Chemigation	Prevathon	Chlorantraniliprole	52.7 (Low)
Chemigation	Prevathon	Chlorantraniliprole	75.1 (High)
Chemigation	Brigade	Bifenthrin	36.8 (Low)
Chemigation	Brigade	Bifenthrin	112.1 (High)

### Aerial Application

Aerial applications were simulated based on droplet size and deposition data from field-based aerial applications. Droplets size data were quantified from the mid-canopy of corn fields after aerial applications were conducted (Souza et al. 2019). The middle of the corn canopy was targeted due to the *S. albicosta* larval feeding activity in the corn ear zone (Montezano et al. 2019a). Combinations of nozzle type and pressure were tested in the wind tunnel and spray chamber and chosen to provide the targeted droplet size to simulate aerial application, following Souza et al. (2019) and Montezano et al. (2019a). Applications were performed using a 2-nozzle research track spray chamber (Generation 4 Research Track Sprayer DeVries Manufacturing, Hollandale, MN) to produce a droplet size of 299 to 346 μm. Two ground spray nozzles, TT110015 (TeeJet Technologies, Spraying Systems Co., Glendale Heights, IL), were used and spaced 0.76 m apart, with an application pressure set at 110 kPa. Four Petri dish bottoms (50 mm diameter × 9 mm height, Pall Corporation, Port Washington, NY) were simultaneously placed in the middle of the 2-nozzle research spray chamber table at 0.56 m below the spray nozzle. Fresh leaves from non-Bt corn (Syngenta NK0760-GT) were collected from the field, cut into discs with a diameter of 3.8 cm, and placed in each Petri dish prior to application. After application, each Petri dish remained open for 30 min to allow complete drying before infestation.

### Chemigation Application

Chemigation was conducted under laboratory conditions using a single-track research spray chamber (Generation III, DeVries Manufacturing, Hollandale, MN). Four corn leaves were placed 50.8 cm below the spray nozzle in a single-nozzle track sprayer. The insecticide application was carried out using a ground spray nozzle HF14015 (Hi-Flow (HF), Pentair Hypro, New Brighton, MN) operating at an application pressure of 207 kPa for 6 min and 25 s, resulting in a rate of 1.57 cm ha^−1^ (0.25 ac-inch). Treated corn leaves were positioned outside the spray chamber for 30 min to ensure complete drying. The treated leaves were processed in the same manner as for aerial application. Four Petri dishes were sprayed for each treatment combination (Table 1), with each Petri dish serving as a replicate.

### Infestation after Insecticide Application

After insecticide application, 4 Petri dishes per treatment were infested with either 20 first-instar, 10 second-instar, or 10 third-instar *S. albicosta* larvae. Larvae were carefully transferred to each Petri dish using a soft camel paintbrush (size 2) (Montezano et al. 2019a). Larval mortality was assessed at 16 and 24 h after infestation for the first and second instars. For the third instar, evaluations were conducted at 16, 24, and 41 h, as older larvae require more time to respond to insecticide active ingredients (Dyer et al. 2013, Coates et al. 2020, Farhan et al. 2022). Larvae that did not move after gentle prodding with a paint brush were considered dead.

### Statistical Analysis

The experiment was designed using a complete randomized block design with 4 replicates. The larval mortality dataset was analyzed with a generalized linear mixed model using PROC GLIMMIX in SAS (Statistical Analysis Software, version 9.4, Cary, NC, USA). In this analysis, the treatment and time variables were considered fixed effects for the evaluated response variable, while replicate was nested within treatment and considered a random effect. To assess the main effects and interactions between treatment and time variables, a repeated measures analysis was employed. Time was treated as a repeated fixed factor to detect changes over time and evaluate treatment effects throughout the experiment.

Mortality rates for each instar were examined at specific time points (16, 24, and 41 h after exposure) using the “SLICE” option (Winer 1962). A binomial distribution was applied to meet ANOVA assumptions and to obtain parameter estimates on the model scale. The means for the response variable were back-transformed for presentation in the figures. For the first and second instars, the analysis considered data at 16 and 24 h post-treatment. For the third instar, data from all 3 time intervals (16, 24, and 41 h post-treatment) were included. By employing these time points as “slices,” it was possible to investigate the impacts of the treatments on larval mortality at each specific time point and identify any significant changes over time. Statistical significance of the effects or the interactions were assessed at α = 0.05 level after adjusting for multiple comparisons using Tukey–Kramer’s adjustment.

## Results

### First Instar

**Fig. 1.**
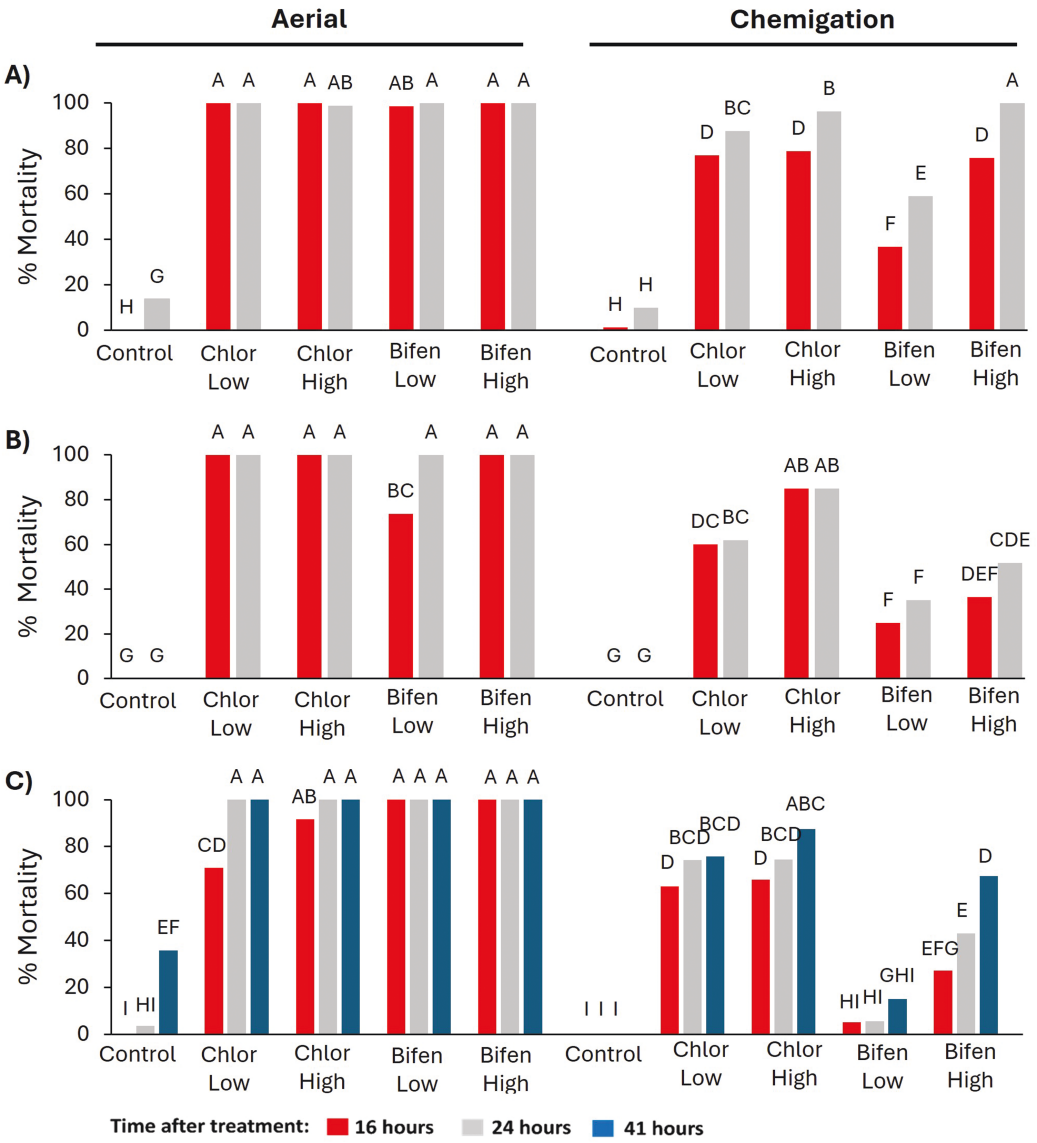
Bar graphs show the percent of mortality for the *Striacosta albicosta* for the A) first, B) second, and C) third instar exposed to chlorantraniliprole and bifenthrin at lower and higher doses after 16 (red), 24 (silver), and 41 (blue) h of exposure under controlled conditions. Each instar was analyzed separately by using repeated measures over time. Treatments with the same letters were not statistically different from each other, after adjusting for multiple comparison through Tukey–Kramer adjustment at significance level α = 0.05. A) Standard error (SE) = 6.012, B) SE = 6.845, C) SE = 12.027. Chlor indicates chlorantraniliprole as the active ingredient, while Bifen indicates bifenthrin as the active ingredient.

There was a significant effect of treatment, time, and the interaction between treatment and time on mortality for the first instar of *S. albicosta* (Table 2 and Table S1). The control groups differed from all insecticide treatments, both for aerial application and chemigation, indicating high susceptibility of *S. albicosta* to insecticides applied under both application methods. Regardless of the evaluation time, chlorantraniliprole and bifenthrin consistently resulted in over 98.6% mortality of *S. albicosta* when aerial application was employed (Fig. 1A).

**Table 2.** Output from repeated measures generalized linear mixed models testing for significant effects of treatment (combination of application types, products, active ingredients, and insecticide rates), time (16, 24, and 41 h), and their interaction on mortality of the first 3 larval instars of *Striacosta albicosta*

Effect	Instar														
		1^st^					2nd					3^rd^			
	SE	DF	Den DF	*F*	*P*	SE	DF	Den DF	*F*	*P*	SE	DF	Den DF	*F*	*P*
Treatment	5.75	9	60	119.27	<0.0001	11.86	9	60	25.00	<0.0001	8.87	9	90	102.42	<0.0001
Time	4.25	1	60	52.04	<0.0001	4.84	1	60	12.13	0.0009	8.50	2	90	14.50	<0.0001
Treatment*time	6.01	9	60	5.06	<0.0001	6.84	9	60	3.52	0.0015	12.03	18	90	2.43	0.0032

*P*-values were considered significant based on α = 0.05 level of significance.

For chemigation, the overall trends indicated an increase in *S. albicosta* mortality from 16 to 24 h after infestation. Moreover, there was a high rate of mortality, with no difference regardless of the evaluation time and label rates for chlorantraniliprole (Fig. 1A). However, it is important to note that a high dose of bifenthrin achieved 100% mortality of *S. albicosta* 24 h after infestation, whereas low rates of bifenthrin at the same time point resulted in 59% mortality (Fig. 1A).

### Second Instar

There was a significant effect of treatment, time, and the interaction between treatment and time on mortality for the second instar of *S. albicosta* (Table 2 and Table S1). For aerial application, all insecticide treatments differed from the control, achieving 100% mortality 24 h after application (Fig. 1B). In addition, even the low bifenthrin application rate resulted in 100% mortality at 24 h after treatment (Fig. 1B).

In contrast, chemigation exhibited consistently lower performance compared to aerial application. Despite an initial statistical difference between low and high chlorantraniliprole rates at 16 h, no difference was observed at the 24 h evaluation time (Fig. 1B). In addition, the high rate of bifenthrin did not show a distinction from the low application rate in terms of mortality of the second instar at 16 h after treatment (Fig. 1B).

### Third Instar

There was a significant effect of treatment, time, and the interaction between treatment and time on mortality for the third instar of *S. albicosta* (Table 2 and Table S1). In general, the results suggest aerial application provides superior control, achieving 100% of mortality of third instar *S. albicosta* larvae after 41 h, compared to chemigation (Fig. 1C). As expected, the control groups were statistically different from all insecticide treatments for aerial application. However, the simulation of aerial application with a low chlorantraniliprole label rate proved to be ineffective, resulting in 70.9% mortality within the first 16 h of evaluation for the third instar of *S. albicosta.* In contrast, chemigation did not show the same trend. In this case, chlorantraniliprole outperformed bifenthrin at a low rate. However, the low rate of chlorantraniliprole and high rate of bifenthrin were not statistically different at 41 h after infestation, achieving 75.8% and 67.5% mortality, respectively. The high label rate of bifenthrin was different from the low rate at 24 and 41 h after infestation. A low bifenthrin rate applied through chemigation was not statistically different from the control group, resulting in only 14.9% mortality 41 h after infestation (Fig. 1C). These results highlight that a low bifenthrin rate applied through chemigation is insufficient to effectively control the third *S. albicosta* instar.

## Discussion

Overall, simulated aerial applications of chlorantraniliprole and bifenthrin were highly effective, causing 100% mortality of first and second instar *S. albicosta* within 24 h and 100% mortality of third-instar larvae within 41 h, regardless of insecticide rate (Fig. 1). Overall, chemigation applications showed highest mortality for high and low rates of chlorantraniliprole and the high rate of bifenthrin. However, mortality was lower as instar increased, with the low rate of bifenthrin performing poorly in some scenarios (Fig. 1). Therefore, to account for potential variability in insecticide susceptibility at different developmental stages, we conducted an independent analysis of the mortality rates of the 3 *S. albicosta* instars, tracking these rates over specific time intervals to determine whether there were any significant changes over time. The effectiveness of treatments applied through chemigation varied depending on larval instar. Thus, these bioassays found that first instars of *S. albicosta* could be successfully controlled by both application methods and insecticides, except for chemigation at the low rate of bifenthrin. However, our results demonstrate that the second and third instars of *S. albicosta* are more challenging to control via chemigation. In these cases, chlorantraniliprole and the high rate of bifenthrin are more likely to provide satisfactory control. The potential for a prolonged oviposition and hatching period following larval dispersal on the corn plant may add more complexity to insecticide and application method selection (Paula-Moraes et al. 2012a, Farhan et al. 2022). Weissling et al. (1992) compared aerial and chemigation applications under field conditions for the European corn borer, *Ostrinia nubilalis* (Hübner), showing that chemigation application of chlorpyrifos greatly reduced the first-generation survival in comparison to aerial application during the whorl-stage of corn (V15-V18). However, our findings do not align with these results, most likely due to the corn stage and exposure of *S. albicosta* larvae that occurs at application time.

The most recent study comparing aerial and chemigation applications for *S. albicosta* management in corn fields was performed in 1984 (Pilcher and Peairs 1984). These findings are less relevant due to product removal from the market and outdated chemigation equipment. A more recent study by Montezano et al. (2019a) assessed the efficacy of bifenthrin in simulating aerial application against the *S. albicosta.* The authors found that commercial insecticides, such as bifenthrin, resulted in 100% mortality of *S. albicosta* from field-collected populations. Our findings corroborate the findings by Montezano et al. (2019a) and demonstrate similar effectiveness when bifenthrin was applied at a rate of 18.7 L ha^−1^ under controlled conditions. Bifenthrin, along with other insecticides from the pyrethroid chemical family, is widely used due to its affordability and market availability, which are critical factors for farmers in making decisions regarding pest management practices in corn (Dewar 2016, Archibald et al. 2018, Montezano et al. 2019a, Souza et al. 2019). Chlorantraniliprole is not as extensively used by farmers in the Midwestern US due to its recent discovery (Jeanguenat 2013, Richardson et al. 2020), higher cost and narrower range of corn pests it can control (Hannig et al. 2009). Conversely, farmers primarily rely on chlorantraniliprole insecticide in Ontario, Canada to protect their corn fields (Farhan et al. 2022). To assess its effectiveness, Farhan et al. (2022) developed susceptibility bioassays to examine the efficacy of chlorantraniliprole on the first and third instars of *S. albicosta.* The results indicated that the first instars of *S. albicosta* were 7 times more susceptible to chlorantraniliprole than to spinetoram active ingredient (IRAC Class 5). On the other hand, third instars were almost 26-fold more susceptible to chlorantraniliprole and lambda-cyhalothrin (IRAC Class 3) compared to spinetoram and methoxyfenozide (IRAC Class 18) (Farhan et al. 2022). Pes et al. (2020) showed that translocation of chlorantraniliprole in corn is greater when applied as a seed treatment compared to foliar application. When chlorantraniliprole was applied by foliar application V3 stage corn, this active ingredient was no longer detected by the V6 stage. It remains a question of whether chlorantraniliprole when applied via chemigation will result in translocation to the target site (tassel) where *S. albicosta* larvae are located. Bifenthrin has contact or ingestion efficacy and cannot be translocated through the plant (Johnson et al. 2010).

Chemigation applications using center pivot systems and sprinkler packages were extensively studied during the 1980s (Pilcher and Peairs 1984, Weissling et al. 1992). Bynum et al. (1991) demonstrated that a high volume of water (21,500 L ha^−1^) applied by an irrigation system for chemigation in corn and sorghum might wash away some of the tracer they used to quantify deposition efficacy. The lower performance of applied insecticides in our study might be due to limited leaf-holding capacity, which means that application of carrier volumes greater than the limited amount of liquid that a leaf is capable of holding will result in run off rather than increased volume at the target site, which supports the findings of Bynum et al. (1991). Similar to our assumptions, Cunha da and Nascimento (2013) confirmed greater active ingredient deposition on corn leaves when an insecticide was applied by aerial application compared to chemigation. These findings corroborate our results for the chemigation application and lower performance of applied insecticides for *S. albicosta* management.

Even though aerial application is the most used method, when corn is fully grown (tasseling stage), insecticide droplet deposition through the corn canopy becomes uneven due to environmental factors, plant morphology, and the nature of the aerial application process. Indeed, results from Bynum et al. (1991) and Weissling et al. (1992) demonstrated that most droplets from aerial application land on the top of leaves near the tassel in corn fields. Conversely, chemigation application has a lower tendency for drift potential, and droplets penetrate more through the canopy (Weissling et al. 1992). In addition, the application methods examined in controlled conditions were used to deliver the spray pattern collected from the mid-canopy of corn. Under these conditions, *S. albicosta* larvae exposed to the treatment could not escape from the sprayed surfaces and Petri dishes, which may differ from real-world field conditions. Furthermore, 3 larval stages (first, second, and third instars) of *S. albicosta* are highly mobile within the corn plant, influencing larval exposure to applied insecticides (Montezano et al. 2019a, Souza et al. 2019). Therefore, when extrapolating results from our study to the field, special caution should be taken when considering larval movement behavior and environmental factors that might influence the efficacy of applied insecticides.

Our study suggests that the simulated aerial and chemigation application methods used in this study for commercially formulated insecticides have the potential to effectively manage *S. albicosta* in corn fields. The effectiveness of applied bifenthrin was high for aerial application for all 3 *S. albicosta* larval stages when environmental factors were excluded. In contrast, low bifenthrin label rates were ineffective in the simulated chemigation application. Our findings show that the same insecticide active ingredients may not achieve the same efficacy when applied using different application methods such as aerial application or chemigation. Special attention should be given to selecting management strategies, insecticide modes of action, and insecticide rates for *S. albicosta* control in the field. The results of this research will be used to conduct studies in the field conditions to generate data that will examine the efficacy and efficiency of insecticides and application methods under more realistic field conditions.

## Supplementary Data

Supplementary data are available at *Journal of Economic Entomology* online.

toae306_suppl_Supplementary_Table_S1
