# Supplementary material for: Comparing simulated aerial and chemigation insecticide applications to manage western bean cutworm (Lepidoptera: Noctuidae) in corn
Source: J Econ Entomol. 2025 Jan 13;118(2):672–9. doi: 10.1093/jee/toae306 (PMC12034310; doi:10.1093/jee/toae306)
Supplement: toae306_suppl_Supplementary_Table_S1 [file toae306_suppl_supplementary_table_s1.docx]

**Table S1**. Covariance parameters from the repeated measures GLMM model used for testing effects of treatment (combination of application types, products, active ingredients and insecticide rates), and time (16, 24 and 41 hours), and their interactions for the first three larval instar of *Striacosta albicosta.* A compound symmetry covariance structure was found to have the best model fit and was used for all three larval instar analyses.

| Covariance Parameter Estimates | | | | | | | |
| --- | --- | --- | --- | --- | --- | --- | --- |
|  |  | 1^st^ instar | | 2^nd^ instar | | 3^rd^ instar | |
|  | Subject | Estimate | Standard Error | Estimate | Standard Error | Estimate | Standard Error |
| Variance | Id(Trt) | 5.3224 | 8.0808 | 7.2153 | 10.4759 | 65.8741 | 22.8721 |
| CS | Id(Trt) | 30.0677 | 11.4975 | 234.37 | 57.8833 | 12.9073 | 15.6507 |
| Residual |  | 30.8170 | - | 39.6345 | - | 78.7814 | - |
